# Review of investigational medical devices' clinical trials and regulations in Africa as a benchmark for new innovations

**DOI:** 10.3389/fmedt.2022.952767

**Published:** 2022-07-28

**Authors:** Brian Matovu, Mercy Takuwa, Charles Norman Mpaata, Fiona Denison, Noah Kiwanuka, Steff Lewis, John Norrie, Sam Ononge, Owen Muhimbise, Sharon Tuck, Maureen Dimitri Etuket, Robert T. Ssekitoleko

**Affiliations:** ^1^Biomedical Engineering Unit, Department of Physiology, School of Biomedical Sciences College of Health Sciences, Makerere University, Kampala, Uganda; ^2^Department of Epidemiology and Biostatistics, The Queen's Medical Research Institute, College of Medicine and Veterinary Medicine, University of Edinburgh, Edinburgh, United Kingdom; ^3^Edinburgh Medical School, Clinical Trials Unit, School of Public Health, College of Health Sciences, Makerere University, Kampala, Uganda; ^4^Usher Institute, Edinburgh Medical School, University of Edinburgh, Edinburgh, United Kingdom; ^5^Department of Obstetrics and Gynaecology, School of Medicine, College of Health Sciences, Makerere University, Kampala, Uganda

**Keywords:** medical devices, clinical trials, medical innovations, medical device regulations, investigational medical devices, health technical assessment

## Abstract

Medical technologies present a huge potential in improving global health playing a key role toward achieving Sustainable Development Goal 3 by 2030. A number of clinicians, innovators, business entities and biomedical engineers among others have developed a number of innovative medical devices and technologies to address the healthcare challenges especially in Africa. Globally, medical devices clinical trials present the most acceptable method for determining the risks and benefits of medical device innovations with the aim of ascertaining their effectiveness and safety as compared with established medical practice. However, there are very few medical device clinical trials reported in Africa compared to other regions like USA, UK and Europe. Most of the medical device clinical trials reported in Africa are addressing challenges around HIV/AIDS, maternal health and NCDs. In this mini review, we report about some of the published medical device clinical trials in Africa PubMed and Google Scholar and their associated challenges.

## Introduction

Africa has been reported to carry about 25% of the world's disease burden (1) and also shares a global health expenditure of <1% (2). This has left many of its citizens with limited access to essential health services and produces few medicines and medical devices consumed on the continent (2). Africa is also observing an increase in the prevalence of non-communicable diseases in the recent years despite the huge burden presented by the traditional infectious diseases such as HIV, tuberculosis (TB), and malaria that have long been the most bulging contributors to the disease burden (2, 3).

To tackle the global health burden in Africa, a number of clinicians, innovators, business entities and biomedical engineers among others have developed innovative medical devices and technologies to address the healthcare challenges. Some of these medical device innovations have been initiated by Africans and others have been through various international collaborations (4). These innovations are at various stages and need to be tested for their safety and efficiency through clinical trials before reaching the market. Globally, medical devices clinical trials present the most acceptable method for determining the risks and benefits of medical device innovations with the aim of establishing their efficacy and safety as compared with established medical practice (5). Effective healthcare delivery through research, innovation and development of medical devices would be constrained if clinical trials were not carried out.

According to the WHO, a medical device and a medical equipment are different. It distinguishes a medical device as an item used in the prevention, diagnosis or treatment of disease, or for detecting, measuring, restoring, correcting or modifying the structure or function of the body for some health benefit. However, a medical equipment excludes implantable, disposable or single-use medical devices (6). Other agencies like the Food and Drug Administration, Health Canada and the United Kingdom's Medicines and Healthcare products Regulatory Agency (MHRA) and many others have adopted similar definitions with slight modifications and this is the same with African regulatory agencies like the National Drug Authority (NDA) of Uganda and the South African Health Products Regulatory Authority (SAHPRA) among others. Furthermore, the Global Harmonization Task Force (GHTF) and its follow-up initiative, the International Medical Devices Regulators Forum (IMDRF), are encouraging the global convergence of regulations and definitions which contributes to a high level of safety protection worldwide and facilitate trade. The IMDRF is advocating for provisions on Unique Device Identification, conformity assessment procedures, technical documentation, general safety and performance requirements, classification rules, and clinical investigations around medical device and innovations (7, 8).

## Classification of medical devices in Africa

Unlike the USA and EU where they have established classifications systems for medical devices, many African countries are facing difficulties in classifying devices arising from the multiplicity of devices, wide application and limited financial and human resource capacity (9). The GHTF Risk Classification of medical devices is based on four classes i.e. Class A, B, C, and D; with Class A representing lowest-risk devices and Class D the highest risk devices. The EU and GHTF Taxonomies are essentially equivalent, both grounded on four classes and these devices are assigned to a class according to their inherent probability to harm the patient, intended application and technology (7).

Many countries in Africa have obtained National Regulatory Authorities or Agencies (NRAs) to regulate medical devices alongside drugs and other food and animal products. There has also been an increase in the number of NRAs since the WHO survey in 2005 in various African countries which found that only 3 countries had an NRA in place, while 29 countries had minimal and 14 no regulations in place at that time (7, 10).

Currently countries like Egypt have the Egyptian Drug Authority (EDA) that classifies medical devices as Class I, IIa, IIb and III while Ethiopia has the Food Medicine and Healthcare Administration and Control Authority of Ethiopia (FMHACA) with a classification system of I, II, III and IV. With this classification system, Class I represents simple devices with history of safe use and have a low risk e.g., the tongue depressor, class IIa represents devices that have a relatively low risk to the human body e.g., the infusion pumps, class IIb represents devices that have a relatively high risk to the human body e.g., hip implants and lastly the class III represents medical devices that may endanger the Patient's life and possess the highest risk e.g., pacemakers.

Other countries like Uganda, Kenya, Tanzania and South Africa have the various NRAs which all classify medical devices as A, B, C and D (7) as shown in Table 1. Class A devices pose the lowest risk to the patient e.g., the wheelchairs and class D being the highest risk to patient and public e.g., the breast implants. The disparities across NRAs come in when some devices fall is different classes dues to the different classification systems e.g., in Uganda, the wheelchair is a class A medical device according to the NDA while in Egypt it's a class IIa medical device according to the EDA. Efforts should be in place to ensure that there is a uniform classification system for innovative medical device trials to be easily carried out across different countries in Africa with a harmonized regulatory framework.

**Table 1:** Medical devices classification systems in some African countries and the responsible National Regulatory Authorities.

**Country**	**Medical devices classification system**	**National Regulatory Authority (NRAs)**
Uganda	A, B, C, D	Uganda National Drugs Authority (NDA)
Egypt	I, IIa, IIb, III	Egyptian Drug Authority (EDA)
Ethiopia	I, II, III, IV	Food Medicine and Healthcare Administration and Control Authority of Ethiopia (FMHACA)
Tanzania	A, B, C, D	Tanzanian Food and Drugs Authority (FDA)
South Africa	A, B, C, D	South African Health Products Authority (SAHPA)
Ethiopia	I, II, III, IV	Food Medicine and Healthcare Administration and Control Authority of Ethiopia (FMHACA)
Zambia	A, B, C, D	Zambia Medicines Regulatory Authority (ZAMRA)
Algeria	I, IIa, IIb, III	Direction de la Pharmacie et du Médicament
Nigeria	A, B, C, D	National Agency for Food and Drug Administration and Control (NAFDAC)
Burkina Faso	No identified classification	Direction Générale de la Pharmacie, du Médicament et des Laboratoires
Burundi	No identified classification	Direction de la pharmacie
South Sudan	A, B, C, D	Food and Drugs Control Authority
Angola	No identified classification	National Directorate for Medicines and Equipment
Benin	No identified classification	Direction de la Pharmacie et des explorations diagnostics
Botswana	A, B, C, D	Drug Regulatory Services
Zimbabwe	No information	Medical Devices Unit, Medicines Control Authority

The various African governments have set up these NRAs to regulate medical devices and other medicinal products, however, they have some limitations like limited human resource especially biomedical engineers who understand medical devices better, few medical device innovators in Africa compared to other regions and varying governments priorities and agendas which do not see medical device regulation as a matter that needs their attention. Some government agencies have put controls on the use and sale of medical devices and ensure that medical devices under research and those on market within their countries' jurisdictions are compliant with national and international standards. For example, when a medical device is innovated and manufactured in Uganda, the innovator or researcher must apply for Institutional Review Board clearance from approved centers and national clearance from Uganda National Council for Science and Technology (UNCST) before starting any clinical testing or trials. Manufacturers must also apply to these bodies to approve use of the device and must provide satisfactory clinical results about their devices.

Various medical devices' innovators struggle to see their innovations come to application and need to work closely with different NRAs in the respective countries, although the regulatory pathway is unclear. Some medical devices if not all, need to go through clinical trials to ascertain their safety and effectiveness but few of these are reported from African countries. Innovators of medical devices also need to know the class of the medical device they are innovating to guide the best clinical trial to be carried out and also to easily navigate the approval processes.

## Selected medical devices clinical trials in Africa

In Africa, a number of medical device innovations have undergone clinical trials to address a number of healthcare challenges in the region and globally. We reviewed clinical trials in Africa published mainly in PubMed and Google Scholar between 2000 and 2021. Some of these trials have been tabulated in Table 2 giving details of the year of publication of the trial, the country where the trial took place, the device name under trial, the classification of that device according to the GHTF, the stage at which the device was tested, the healthcare challenge being addressed and the type of device under trial. The devices listed in this mini review address challenges concerning non-communicable diseases, maternal and child health, infectious diseases, drug delivery and male circumcision among others as discussed below. We see a high number of trials published after 2010 compared to before that year.

**Table 2:** Listing of some of the medical devices' clinical trials that have taken place in Africa showing the year of publication, the country where the trial took place, the device name, the class of the device according to the GHTF, the stage of the device under trial, the medical challenge being addressed and the type of medical device.

**Paper**	**Authors and year of publication**	**Country**	**Device name**	**Class of medical device according to the GHTF**	**Stage of medical device**	**Medical challenge addressed**	**Type of medical device**	**Reference**
Non-pneumatic anti-shock garment (NASG), a first-aid device to decrease maternal mortality from obstetric hemorrhage: a cluster randomized trial	Magwali et al., 2013	Zimbabwe	Non-Pneumatic Anti-Shock Garment (NASG)	A	Market	Postpartum Hemorrhage	Prevention	(11)
Absorbable vs. silk sutures for surgical treatment of trachomatous trichiasis in Ethiopia: a randomized controlled trial	Rajak et al., 2011	Ethiopia	Absorbable vs. silk Sutures	D (Absorbale) C (Silk Sutures)	Market	Trachomatous trichiasis	Treatment	(12)
Assessing the role of the non-pneumatic anti-shock garment in reducing mortality from postpartum hemorrhage in Nigeria	Ojengbede et al., 2011	Nigeria	Non-Pneumatic Anti-Shock Garment (NASG)	A	Market	Postpartum Hemorrhage	Prevention	(13)
Implementation and operational research: a randomized noninferiority trial of accucirc device vs. mogen clamp for early infant male circumcision in Zimbabwe	Mavhu et al., 2015	Zimbabwe	AccuCirc device vs. Mogen Clamp	B	Market	HIV	Prevention	(14)
Quality of induced sputum using a human-powered nebuliser in a mobile human immunodeficiency virus testing service in South Africa	Kranzer et al., 2011	South Africa	Human powered Nebuliser	B	Market	HIV testing	Treatment	(15)
Difference in blood pressure readings with mercury and automated devices: Impact on hypertension estimates in Dar es Salaam, Tanzania	Chiolero et al., 2016	Tanzania	Mercury vs. automated blood pressure devices	B	Market	Hypertension	Diagnostic	(16)
Accuracy assessment of a novel blood pressure measurement device in a South African adult population: Tensoval duo control	de Greeff A et al., 2011	South Africa	Tensoval duo control blood pressure device	B	Market	Hypertension	Diagnostic	(17)
Effect of a novel vital sign device on maternal mortality and morbidity in low-resource settings: a pragmatic, stepped-wedge, cluster-randomized controlled trial	Vousden et al., 2019	Harare | Karnataka	CRADLE Vital Sign Alert	B	Market	Maternal mortality and morbidity	Diagnostic	(18)
Use of the ShangRing circumcision device in boys below 18 years old in Kenya: results from a pilot study	Awori et al., 2017	Kenya	ShangRing Circumcision device	B	Market	HIV	Prevention	(19)
Intermittent fetal heart rate monitoring using a fetoscope or hand held Doppler in rural Tanzania: a randomized controlled trial	Mdoe et al., 2018	Tanzania	Fetoscope	B	Market	Neonatal Mortality	Diagnostic	(20)
Effects of the copper intrauterine device vs. injectable progestin contraception on pregnancy rates and method discontinuation among women attending termination of pregnancy services in South Africa: a pragmatic randomized controlled trial	Hofmeyr et al., 2016	South Africa	Copper intrauterine device vs. injectable progestin contraception	D	Market	Unwanted pregnancy	Prevention	(21)
Host markers in QuantiFERON supernatants differentiate active TB from latent TB infection: preliminary report	Chegou et al., 2009	South Africa	QuantiFERON TB Gold In Tube (QFT)	B	Market	Tuberculosis	Diagnostic	(22)
Safety and efficacy of an intraocular Fresnel prism intraocular lens in patients with advanced macular disease: initial clinical experience	Potgieter et al., 2014	South Africa	Intraocular Fresnel prism intraocular lens	C	Market	Advanced Macular Disease	Treatment	(23)
Rapid, minimally invasive adult voluntary male circumcision: a randomized trial of Unicirc, a novel disposable device.	Millard et al., 2014	South Africa	Unicirc vs. Surgical Circumcision	B	Market	HIV	Prevention	(24)
Clinical trials using the Shang Ring device for male circumcision in Africa: a review	Barone, et al., 2014	Kenya, Uganda, Zambia	Shang Ring Device	B	1st Proof of concept in China 2005, then in Kenya 2009 and Full device in Uganda and Zambia	Male Circumcision, HIV/AIDS, HSV-2	Prevention	(25)
Evaluation of a rapid diagnostic test in the diagnosis of toxoplasmosis in pregnant women in Cotonou (Bénin)	Ogouyèmi-Hounto et al., 2014	Benin	ImmunoComb^®^ Toxo IgG and ImmunoComb^®^ Toxo IgMassays	A	Market	Toxoplasmosis in pregnant women	Diagnostic assay	(26)
Sutureless adult voluntary male circumcision with topical anesthetic: a randomized field trial of unicirc, a single-use surgical instrument	Shenje et al., 2016	South Africa	Unicirc Device	B	second prototype/version	Male Circumcision, HIV/AIDS, HSV-2	Prevention	(27)
Effect of the CRADLE vital signs alert device intervention on referrals for obstetric hemorrhage in low-middle income countries: a secondary analysis of a stepped- wedge cluster-randomized control trial	Giblin et al., 2021	Africa (Mulago Kampala), Asia and India	The CRADLE (Community blood pressure monitoring in Rural Africa & Asia: Detection of underLying pre-Eclampsia and shock) Vital Signs Alert device (CRADLE VSA) is a semi-automated vital signs measurement device	B	Fully functional device	Preeclampsia	Diagnostic	(28)
The diagnostic accuracy of urine-based Xpert MTB/RIF in HIV-infected hospitalized patients who are smear- negative or sputum scarce	Peter et al., 2012	South Africa	Urine-Based Xpert MTB/RIF	B	Fully functional device	HIV, TB, AMR	Diagnostic	(29)
PrePex circumcision surveillance: adverse events and analgesia for device removal	Lebina et al., 2018	South Africa	PrePex device	B	Fully functional device	Male Circumcision, HIV/AIDS, HSV-2	Prevention	(30)
Institutionalizing postpartum intrauterine device (IUD) services in Sri Lanka, Tanzania, and Nepal: study protocol for a cluster randomized stepped-wedge trial	Canning et al., 2016	Sri Lanka, Tanzania, Nepal	Intrauterine Device (IUD)	D	Market	Postpartum contraception use	Prevention	(31)
Clinical field testing of trans-femoral prosthetic technologies: sresin-wood and ICRC-polypropylene	Jensen et al., 2004	Tanzania	Transfemoral prosthetic systems	A	Market	Lower Limb disability	Rehabilitaion	(32)
Neonatal resuscitation using a laryngeal mask airway: a randomized trial in Uganda	Pejovic et al., 2018	Uganda	i-gel	B	Market	Neonatal Resuscitation	Treatment	(33)
Cost-effectiveness of the non-pneumatic anti-shock garment (NASG): evidence from a cluster randomized controlled trial in Zambia and Zimbabwe	Downing et al., 2015	Zambia and Zimbabwe	Non-pneumatic anti-shock garment (NASG)	A	Market	Postpartum hemorrhage	Prevention	(34)
Single-Arm Evaluation of the AccuCirc Device for Early Infant Male Circumcision in Botswana	Plank et al., 2014	Botswana	AccuCirc	B	Pilot	Early infant male circumcision	Prevention	(35)
Intracesarean insertion of the Copper T380A vs. 6 weeks post-cesarean: a randomized clinical trial	Lester et al., 2015	Uganda	Copper T380A IUD	D	Market	Contraception	Prevention	(36)
Humid vs. dry incubator: a prospective, randomized, controlled trial	Fawzy et al., 2017	Egypt	Benchtop incubator	B	Market	Human embryo development *ex-vivo*	Treatment	(37)
Bakri balloon vs. condom-loaded Foley's catheter for treatment of atonic postpartum hemorrhage secondary to vaginal delivery: a randomized controlled trial	Darwish et al., 2017	Egypt	CONDOM-loaded Foley's catheter vs. Bakri Balloon	B	Market	Post-partum hemorrhage management	Treatment	(38)
Accuracy of fluid delivery devices for the neonate: are the measures assured?	Okoro et al., 2020	Nigeria	Fluid delivery devices (Infusion giving set, Burette giving set, and blood giving set)	B	Market	Drug delivery	Treatment	(39)
A phase II randomized controlled trial comparing safety, procedure time, and cost of the PrePex™ device to forceps guided surgical circumcision in Zimbabwe	Tshimanga et al., 2016	Zimbabwe	PrePex™	B	Phase II	HIV	Prevention	(40)
The Role of the Nonpneumatic Antishock Garment in Reducing Blood Loss and Mortality Associated with Post-Abortion Hemorrhage	Manandhar et al., 2015	Egypt, Nigeria, Zambia, and Zimbabwe	Non-pneumatic anti-shock garment (NASG)	A	Market	Post-partum hemorrhage	Prevention	(41)
AutoSyP: a low-cost, low-power syringe pump for use in low-resource settings	Juarez et al., 2016	Malawi	AutoSyP	B	Pilot	Drug delivery	Treatment	(42)
Safety and continued use of the levonorgestrel intrauterine system as compared with the copper intrauterine device among women living with HIV in South Africa: a randomized controlled trial	Todd et al., 2020	South Africa	levonorgestrel intrauterine system as compared with the copper intrauterine device	B	Market	HIV and contraception	Prevention	(43)

Further referring to Table 2, most of the devices (26 devices) were clinically tried when they are already on the market compared to a few that were still in prototyping stage and or in the premarket stage of the innovation design cycle. Also, majority were under class B (21 devices) classification basing on the GHTF classification system as compared to the 6 under class A, 2 under class C and 5 under class D. Probably the devices that were tested when they were already on market might have had their clinical trials outside Africa while still in the premarket stage. The high number of devices in class B could be related to the ease to use and innovate such devices and hence less cost in development and implementation compared to the class D devices with a highest risk to the patient.

## Devices for maternal and child health

Sub-Saharan Africa (SSA) still has the highest maternal and child deaths in the world despite numerous efforts to reduce the high mortality and morbidity (44). Some medical devices have been reported about through clinical trials in addressing maternal and child health challenges in Africa for example the Non-Pneumatic AntiShock Garment (NASG) which is an innovation developed as a first aid tool to combat postpartum hemorrhage (PPH), one of Africa's leading causes of maternal death. This was trialed by Ojengbede et al. in 2011 in Nigeria (13); by Miller et al. in 2013 in Zimbabwe (11); by Magwali et al. in 2013 in Zimbabwe; and lastly by Downing Jannelle in 2015 in Zambia and Zimbabwe (34). All these studies were carried out to determine whether application of the NASG at the clinic level in comparison to the referral hospital level lowered maternal deaths as well as recovery time from shock resulting from severe Obstetric Hemorrhage and to ascertain the safety of the NASG when used at the clinic level. Ojengbede et al. in 2011 aimed at determining whether NASG showed potential for reducing PPH mortality in Nigerian referral facilities (13).

Furthermore, Vouseden and Giblin et al. in 2019 and 2021 reported results from the CRADLE Vital Signs Alert device (CRADLE VSA) as a semi-automated device designed for use in resource limited environments to detect underlying preeclampsia and hysterectomy in Harare Zimbabwe (18, 28). This device Measures a patient's heart rate and blood pressure and calculates the shock index (ratio of heartrate to the systolic blood pressure) before displaying a signal light alert depending on shock index thresholds. The CRADLE VSA device recognizes patients who need to be referred for hemorrhagic shock, which would be useful in low-income countries where decisions are could be made by inexperienced healthcare practitioners.

In 2018, Mdoe et al. reported about a clinical trial for a fetoscope or a hand held Doppler for intermittent fetal heart rate monitoring in rural Tanzania (20). The rate of abnormal fetal heart rate (FHR) detection and adverse perinatal outcomes were examined in this study among women monitored periodically by Doppler or fetoscope in a rural resource-limited setting. This study found no statistically significant difference between occasionally used Doppler and fetoscope in detecting abnormal FHR or unfavorable neonatal outcomes (20).

## Devices for circumcision and HIV/AIDS

Some medical devices have been developed and clinically tested in various African countries to address HIV/AIDS epidemic mainly focusing on circumcision. Among these include the AccuCirc Device, ShangRing device, UniCirc device and the Prepex device for circumcision. In 2014, Plank et al. reported about a pilot study on the AccuCirc in Botswana to evaluate device safety and parental satisfaction (35). In 2015 in Zimbabwe, Mavhu reported a randomized non-inferiority trial conducted to determine the safety and acceptability of the same device with the Mogen clamp (35). The device is intended for early infant male circumcision as a possible HIV prevention strategy (14).

A ShangRing device for circumcision was tested in Kenya, Uganda and Tanzania in 2014 whose first proof of concept in China (2005), with other trials reported in Kenya in 2009 and 2019 and subsequent years in Uganda and Zambia (19, 25). He further reported that the ShangRing device eliminates need for suturing, it is safe and easy to use and train on. A proof of concept study, an investigation on delayed Shang Ring removal, two studies examining Shang Ring circumcision vs. conventional surgical techniques, and a large field study to assess the safety of Shang Ring circumcision under normal care provision have all been conducted on this device (25).

The Unicirc device was developed in South Africa and has also had a number of clinical trials to ascertain its safety and effectiveness e.g., in 2014 Millard et al. carried out a trial in South Africa, to make comparisons between open surgical circumcision with suturing, and the Unicirc disposable instrument including tissue adhesive (24). Also in 2016, Shenje et al. did a randomized field trial in South Africa to determine the time of healing after device use, pain levels, device safety and the cosmetic results of the penis after circumcision (27).

Lastly, the PrePex device is one of the most reported circumcision devices that have had trials in Africa e.g., in 2016 there was In Zimbabwe, a phase II randomized controlled trial compared the PrePex device to Forceps Guided Surgical Circumcision in terms of safety, procedure time, and cost (40). The results of this trial show that the PrePex method is quick, efficient, and effective, taking about a third of the time of a surgical operation. Also in 2018, Libina et al. conducted a study to determine the safety of PrePex and if analgesia given prior to removal lowers pain in participants (30).

## Other medical devices trials

Other medical device clinical trials reported in Africa include an RCT in Ethiopia where absorbable sutures were compared to silk sutures for surgical intervention in trachomatous trichiasis (12); an RCT for a human powered nebulizer to evaluate the quality of induced sputum in a South African mobile HIV testing service (15); a comparative study in Dar es Salaam, Tanzania in 2006, to distinguish between mercury and automated blood pressure readings and their impact on hypertension estimates (16); the Tensoval duo control blood pressure device in South Africa whose trial was to determine its accuracy of blood pressure measurement among a South African adult population (17); a pilot study of an AutoSyP device which is a cheap, power-efficient syringe pump for use in resource constrained settings like Malawi (42) and in an RCT in Egypt, the Bakri balloon was compared to a condom-loaded Foley's catheter for the treatment of atonic postpartum hemorrhage caused by vaginal delivery (38).

Less device trials have been reported on challenges around rehabilitation and assistive technology and purely laboratory-based devices in Africa. Majority of the device trails are reported from South Africa compared to the countries like Uganda, Zimbabwe, Nigeria, Egypt and Tanzania among others. A further investigation into the disparity between these African countries in conducting clinical trials for medical devices needs to be carried out.

## Challenges affected by medical devices clinical trials in Africa

Generally, Investigational medical devices are essential to the improvement of healthcare. While many devices have significantly enhanced clinical outcomes, most of them require scrutiny and analysis before use as some front great health risks. For a long time, new medical devices have been adopted with minimum scientific evidence to support their use owing to the limited regulatory authorities in African countries. This is because of lack of regulatory bodies with knowledge and experience to regulate the entry and testing of medical devices (45) despite the proper and well streamed regulatory procedures for pharmaceutical products (46).

Currently, most African governments have developed regulatory bodies, such as the Uganda National Drugs Authority in Uganda (47) and South African Health Products Authority (48) in South Africa, to ensure the protection and efficiency of new medical devices. Such bodies are required to act in a well-organized and appropriate style so as to save and benefit patients. Nevertheless, still most African countries do not have well streamed procedures by which new high-risk medical devices are translated from bench to bedside. Instead, most of them rely on imports and hardly do they get to look at investigation results and reports of the devices where the testing was carried out from. It is also true that there is quick approval granted to the low risk devices because they pose low risk to patient health.

In order to increase patient safety and avoid any legal liabilities during medical device usage, there is a need for thorough regulations and adequate pre-marketing data for high and medium risk medical devices. The pre-marketing evaluation and authorization of high and medium risk medical devices ought to be joined with continuous post-marketing surveillance to guarantee benefits and minimize harms of device use in (46, 49). There is need to continuously review medical device regulations for countries that have them and also for those that don't have them to adopt them so as to ensure the safety of consumers. These should be synchronization of the global regulations to accelerate the growth and development of the medical device industry and ensure the establishment of radical medical devices that profit patients minus causing difficulties. The purpose of global harmonization is to decrease regulatory disparities globally, eradicate extreme or country specific requirements, and construct a reliable and transparent universal regulatory organization system (50).

According to Rugera et al. report in 2014, all East African Community member States demonstrated to have one or more regulatory bodies for medical products with the exclusion of Rwanda that had activated a taskforce to manage the development and establishment of a Food and Drug Authority (51). Additionally, he reported that the capability to control both medical devices and diagnostic tests inside the EAC is inadequate and Formal Technology Assessment Programs are deficient in all Partner States. The experiences among the EAC member states is not different across the rest of Africa where regulation of medical devices is weak, it is an abandoned sector and there is reduced capability to do so (51).

Other challenges around medical devices clinical trials are not limited to inadequate funding, limited expertise around device clinical trials, few medical device innovators and high gap between academia, research and industry in most African countries. Few biomedical engineers and other personnel competent in medical devices and their regulation are available on the African continent. This poses a great challenge on how the NRAs can regulate such a medical devices' sector in addition to the few medical devices innovators available on the continent. The processes and systems for translating medical devices from idea to market are not clear and hence many potential innovators are left blinded on where to seek support whether financial or technical.

## Conclusion

Not many African countries have put in place measures and regulations to streamline the medical devices clinical trials. These challenges of limited financial capacity, human resource, operational barricades, unclear ethical and regulatory pathways, competing demands and lack of a research atmosphere are still affecting the investigational medical devices clinical trial in Africa because this is a neglected area for now in many of these countries. Medical devices' clinical trials that target specific healthcare diseases and conditions affecting mainly people in Africa are logically best carried in these countries.

Despite all that, many African countries have improved their research output and medical innovators are being mentored and facilitated to address the various healthcare challenges affecting the continent. This is shown by the increasing trend of funding to Africa for various medical device innovations which later will reach clinical trials before hitting the market. Expertise and resources are being put together as many Institutional review boards are being facilitated to approve medical device clinical trials.

Lastly, researchers, academicians and policy makers in Africa should take a vested interest in carrying out Health Technology Assessment (HTA) to ascertain the properties (medical, social, ethical and economical), effects and impact of the various technologies on their healthcare systems. This will increase the visibility of medical devices research and attract more work on device trials and foster a better environment for innovators on the continent. A deeper investigation into medical device clinical trials especially those registered with clinicaltrials.gov and the pan African clinical trials register should also be carried out to provide a bigger understanding on the status of medical device's research in Africa.

## Author contributions

BM, MT, CM, and RS conceptualized the manuscript, performed the literature search, and putting together the draft versions. FD, NK, SL, JN, OM, ST, and ME supervised and revised the manuscript drafts prepared. All authors contributed to the article and permitted the submitted version.

## Funding

This work was supported in part by the Medical Research Council UK through the grant project Capacity building for a Centre of Design, Innovation and Translational Excellence (CITE) for clinical trials of healthcare technologies in SS Africa (Grant Number: MR/T03937X/1).

## Conflict of interest

The authors declare that the research was conducted in the absence of any commercial or financial relationships that could be construed as a potential conflict of interest.

## Publisher's note

All claims expressed in this article are solely those of the authors and do not necessarily represent those of their affiliated organizations, or those of the publisher, the editors and the reviewers. Any product that may be evaluated in this article, or claim that may be made by its manufacturer, is not guaranteed or endorsed by the publisher.
